# A New Anatomy of Autophagic Clearance: On the Roles of Intrinsic Disorder in the Membrane-Less on Membrane-Encapsulated Mechanism

**DOI:** 10.3390/membranes16070234

**Published:** 2026-07-06

**Authors:** Vladimir N. Uversky, Hana Popelka, Daniel J. Klionsky

**Affiliations:** 1 Department of Molecular Medicine, Morsani College of Medicine, University of South Florida, Tampa, FL 33620, USA; 2 USF Health Byrd Alzheimer’s Research Institute, Morsani College of Medicine, University of South Florida, Tampa, FL 33620, USA; 3 Life Sciences Institute, University of Michigan, Ann Arbor, MI 48109, USA; klionsky@umich.edu

**Keywords:** autophagy, intrinsically disordered protein, intrinsically disordered region, liquid-liquid phase separation, macroautophagy, microautophagy, posttranslational modification, protein1protein interaction

## Abstract

Autophagy is a carefully regulated catabolic process that utilizes assemblies of specific sets of macromolecules operating at multiple stages of the pathway. Discoveries in recent years show that autophagy markedly relies on liquid-liquid phase separation (LLPS). Here, we present parameters that indicate the plasticity of autophagy proteins and their probability to undergo LLPS in macroautophagy and microautophagy. We show that microautophagy is an extremely LLPS-friendly pathway. Several mechanisms involving proteins in the autophagy machinery that drive LLPS on various types of membranes to regulate this process or that undergo LLPS as autophagic cargo are described in detail. We also summarize the factors that modulate the LLPS potential of autophagy proteins. A high probability of autophagy-related proteins to undergo spontaneous LLPS shown here can direct future research on the role of protein droplets in autophagy.

## 1. Introduction

Intrinsically disordered proteins (IDPs) and hybrid proteins with ordered domains and intrinsically disordered regions (IDRs), the prevalence and biological importance of which were recognized around the turn of the 21st century [1,2,3,4], continue to defy classical cellular “norms”. The understanding of the role of these domains started with the fundamental shift from the rigid “lock-and-key” sequence-structure-function paradigm to a “protein structure-function continuum” model that marked a major evolution in molecular biology, moving from a binary view of protein functionality (ordered = active, disordered = inactive) to an inclusive model that recognizes conformational dynamics and disorder as essential constituents of protein function and where structure, disorder, and function are melted together into a “protein trinity” framework defining a protein’s biological activity. In other words, IDPs/IDRs are not “broken” versions of folded proteins, and disorder in proteins is not a lack of organization, but rather a different form of functional information encoded within the amino acid sequence that allows for dynamic interactions and signaling [1,2,3,5,6,7,8,9,10].

Next, it became clear that IDPs/IDRs are characterized by high information density, as, due to their lack of stable structure, almost every amino acid in an IDP/IDR can be used as a functional motif or a site for posttranslational modifications (PTMs) [11,12,13,14,15,16,17,18,19,20,21]. In fact, this informational density is reflected in remarkable spatio-temporal heterogeneity of proteins, where structurally the whole protein can range from completely structure-less, coil-like conformational ensembles to compact (but still highly dynamic) molten globular ensembles, to proteins with a hybrid structure containing both ordered and disordered regions [22], and where even a single protein molecule can have fragments with different structural complexity and folding complicity. This creates a highly dynamic mosaic organization consisting of structural elements such as foldons (independently foldable protein segments), inducible foldons (disordered segments that fold into a fixed structure only upon binding to a specific partner), inducible morphing foldons (promiscuous disordered regions that shape-shift into different structures depending on which partner they bind to), semi-foldons (protein segments trapped in a perpetual state of partial folding, maintaining a loose, transient structure), non-foldons (permanently unstructured regions that remain fully chaotic and flexible), and unfoldons (specific ordered regions that must temporarily unfold to switch the protein into its active state) [22,23,24], with these differently (dis)ordered parts of a protein molecule possessing different functions thereby defining the global protein multifunctionality [23,24,25,26].

This fundamental “rigid to disordered” shift in the understanding of protein functionality was followed by the recognition that IDPs and IDRs are not rare exceptions, but are abundantly present across all proteomes [2,27,28,29,30,31,32,33], that many proteins are functional precisely because they are flexible and lack stable structures [1,3,5,6,7,8,9,34,35,36,37,38,39,40], and that the levels of IDPs/IDRs in proteomes are correlated with the evolutionary complexity of the organisms, where the more advanced species have higher IDP/IDR counts [27,28,30,32,33,41].

Furthermore, acceptance of the intrinsic disorder phenomenon has opened a door to a multiverse of protein interactions, moving from classical “lock-and-key” binding to functional disorder-to-order transitions, high specificity-low affinity signaling interactions, one-to-many and many-to-one binding modes, moonlighting functions, the “fly-casting” mechanism, the formation of fuzzy complexes (where substantial disorder is preserved in the bound state), dynamic scaffolding (i.e., the transient, regulated assembly of signaling proteins by scaffold proteins, which act as mobile, flexible platforms rather than static anchoring structures), and “hot potato” interaction mechanisms (where a partner is rapidly passed between different proteins) [8,35,40,42,43,44,45,46,47,48,49,50,51,52,53,54,55].

Subsequently came a recognition that IDPs/IDRs are commonly involved in the pathogenesis of various human diseases [56,57,58,59,60,61,62,63,64,65,66,67,68,69,70,71,72,73,74,75]. In fact, while these proteins are crucial for normal cellular regulation, signaling, and recognition, their malfunction, often resulting from the failure of protein homeostasis (proteostasis) as reflected in misfolding, aggregation, incorrect signaling and transport, incorrect processing, impaired degradation, aberrant alternative splicing, and aberrant PTMs, is strongly linked to several major pathologies, such as neurodegeneration, cardiovascular disease, cancer, and diabetes. The recognition of the high therapeutic potential of manipulating IDPs/IDRs resulted in a shift of the drug discovery focus from the structure-based rational drug design of the “lock-and-key” type (where small molecules are docked into rigid, well-defined binding pockets) to searches for drugs targeting “protein clouds”, which, for a long time, were considered undruggable [60,76,77,78,79,80,81,82,83,84,85,86,87,88,89].

More recently, protein intrinsic disorder was linked to the liquid-liquid phase separation (LLPS) phenomenon that relies on weak multivalent interactions and drives the biogenesis of various membrane-less organelles and biomolecular condensates [13,90,91,92,93,94,95,96,97,98,99,100]. It was also revealed that LLPS can happen in 1D (e.g., on cytoskeleton or chromatin), 2D (on or within the membrane), and 3D (bulk solution of the cytoplasm or nucleoplasm) [101,102]. The 1D and 2D LLPS are also known as surface-assisted or scaffolded LLPS [103,104,105,106] and can serve as a potential means for cellular signal transduction, where the LLPS propagation has an autowave character allowing for rapid, low-energy cellular adjustments to environmental changes [101,102]. Because LLPS is a reversible temporary process, it is particularly useful in regulation where IDRs play a dominant role. A pathway that utilizes numerous IDRs and relies on tight regulation is autophagy.

In eukaryotic cells, autophagic clearance is a foundational catabolic process that acts as a recycling system to maintain cellular health. Autophagy is the process of self-eating, by which cells degrade and recycle cytoplasmic components to maintain their homeostasis, and represents a complex regulatory network mediated by a broad range of protein-protein interactions [107]. Depending on the mechanisms of sequestration, autophagy is divided into three primary types, macroautophagy, microautophagy, and chaperone-mediated autophagy (CMA), and can be further distinguished by the specificity of its substrates (selective vs. non-selective) [108,109,110,111].

The most robust form of autophagy is macroautophagy, which works by sequestering dysfunctional components, such as damaged proteins, harmful pathogens, and dysfunctiotional or superfluous organelles, within double-membraned vesicles known as autophagosomes. These vesicles subsequently fuse with lysosomes (vacuoles in yeast and plants), where the sequestered materials are degraded by hydrolases and recycled into nutrients for cellular survival [112,113,114]. Macroautophagy is a complex, multistage process with major steps including induction, cargo recognition and packaging, vesicle nucleation, vesicle expansion and completion, Atg protein cycling, vesicle fusion with the vacuole/lysosome, vesicle breakdown, and recycling of the resulting macromolecules [111,115]. Macroautophagy operates via a core set of autophagy-related (ATG) proteins that are conserved across species, ranging from yeast to mammals [116,117], suggesting that autophagy represents a primordial eukaryotic trait that likely emerged during eukaryogenesis and was inherited by all modern eukaryotes [118]. Depending on their functions within the autophagy machinery, the core ATG proteins in yeast/human are grouped into five categories [115,118,119]:

- Initiation Complex: The Atg1/ULK1 complex (Atg1, Atg13, Atg17-Atg31-Atg29 trimer, Atg11, Atg20, and Atg24 in yeast; ULK1, ULK2, ATG13, RB1CC1, and ATG101 in humans);
- Nucleation Unit: Class III PtdIns3K complex I, which generates PtdIns3P (Vps30/Atg6, Vps34, Atg14, Atg38, and Vps15 in yeast; BECN1, PIK3C3/VPS34, PIK3R4/VPS15, ATG14, NRBF2, and AMBRA1 in humans);
- Atg9/ATG9 Trafficking System for membrane supply: Atg9/ATG9A-containing vesicles that act as membrane donors (Atg9, Atg2, Atg18 in yeast; ATG9A, ATG9B, ATG2A, ATG2B, WIPI1, WIPI2, WDR45B/WIPI3, and WDR45/WIPI4 in humans);
- Tethering Factors involved in expansion: Atg2-Atg8 or ATG2-WIPI complex that in humans consists of ATG2A or ATG2B, and one of the four WIPI-type proteins (WIPI1, WIPI2, WDR45B/WIPI3, and WDR45/WIPI4), which facilitate lipid transfer from the ER;
- Two Conjugation Systems: the Atg12/ATG12 system that includes Atg12, Atg5, Atg7, Atg10, and Atg16 in yeast and ATG12, ATG5, ATG7, ATG10, and ATG16L1 (or ATG16L2) in humans; and the Atg8-family system that includes Atg8, Atg7, Atg3, and Atg4 in yeast and LC3A, LC3B, LC3C, GABARAP, GABARAPL1, and GABARAPL2/GATE-16 (ATG8s), ATG7, ATG3, and ATG4 [A, B, C, and D isoforms]) in humans.

Many other proteins, including VMP1 and TMEM41B, fall outside these specific categories but remain essential for the autophagy process playing crucial, independent roles [118,120]. These ATG proteins establish a complex network, interacting with various cytoplasmic components involved in diverse cellular pathways, rendering autophagy a highly intricate process [121]. Although there are ~20 core human ATG proteins that make up the essential machinery for autophagosome formation, based on the proteomic analysis, it was shown that basal autophagy in human cells relies on a complex autophagy interaction network (AIN) containing 409 proteins linked by 751 interactions [122].

In microautophagy, the lysosomal/vacuolar membrane directly invaginates, protrudes, or septates to engulf small portions of the cytoplasm, leading to the direct uptake of the cargo into the lysosomal lumen [123]. Unlike macroautophagy, this process does not rely on the formation of the double-membrane autophagosomes [124]. Instead, this specific process of the direct engulfment of cytosolic cargo via vacuolar/lysosomal membrane invagination starts with the formation of the autophagic tube that invaginates the membrane to sequester cargo, followed by vesicle formation at the tip of the tube that buds into the lumen and is subsequently degraded [125]. Formation of the autophagic tubes, which are specialized, protein-depleted membrane protrusions that extend into the lysosomal/vacuolar lumen and pinch off to form single-membrane vesicles, is regulated by the autophagy pathway [125], which, in yeast, involves the Vacuolar Transporter Chaperone (VTC) complex functioning as a calmodulin (CALM) target to stimulate tonoplast invagination and vesicle scission [126,127]. In humans, the process depends on the Endosomal Sorting Complexes Required for Transport (ESCRT) machinery, which handles the membrane scission required to internalize the cargo without compromising the lysosome’s integrity [128]. Furthermore, several ATG proteins are involved in specific mammalian microautophagy pathways. For example, LC3 is involved in cargo recognition for the selective endosomal microautophagy of receptors, such as SQSTM1/p62 and NBR1; ATG5 and ATG7 are required for certain types of microautophagy, such as the degradation of the STING1 protein or ER remodeling; and RB1CC1/FIP200 and PIK3C3/VPS34 are critical for iron starvation-induced micro-ferritinophagy) [129].

CMA is a highly selective process, where the chaperone HSPA8/Hsc70 recognizes specific cytosolic proteins containing a KFERQ-like motif [130,131,132]. These target proteins are delivered to the lysosomal membrane as part of the HSPA8-substrate complex, along with co-chaperones such as DNAJ/Hsp40, HSP90, and ST13/Hip-STIP1/Hop, where they interact with the cytosolic tail of the lysosome associated membrane protein 2A (LAMP2A) [133,134,135]. In its inactive form, LAMP2A is a monomer, but substrate binding triggers its controlled multimerization into a high-molecular-mass protein complex (~700 kDa) that forms the translocation pore. This 700-kDa multimeric complex forms the actual, transient translocation pore, acting as a channel for substrate uptake [136,137]. Importantly, successful translocation of a substrate requires its complete unfolding. This crucial step is assisted by both the external HSPA8 and a specialized resident isoform, lys-HSPA8, located inside the lysosomal lumen. Lys-HSPA8 is indispensable for CMA, acting to pull the unfolded protein through the LAMP2A channel into the lumen [138,139,140].

Although macroautophagy and microautophagy typically occur in a non-selective manner and are used to degrade cytoplasm in bulk to generate nutrients during starvation, and these processes can also be highly selective allowing cells to specifically target damaged components, aggregated proteins, or specific organelles for degradation [141,142,143,144]. In selective autophagy, particular substrates are recognized and targeted for degradation by specific “cargo receptors”, with specific material removed by the corresponding pathways giving rise to their names. Some of the selective autophagy pathways are aggrephagy (selective removal of protein aggregates), ERphagy/reticulophagy (selective degradation of parts of the endoplasmic reticulum), lipophagy (selective breakdown of lipid droplets to release lipids), lysophagy (targeted removal of damaged lysosomes), mitophagy (selective degradation of damaged or excessive mitochondria), nucleophagy (selective degradation of nuclear components or portions of the nucleus), pexophagy (selective degradation of peroxisomes), ribophagy (selective degradation of ribosomes), and xenophagy (elimination of intracellular pathogens, such as bacteria and viruses) [145,146,147,148,149,150]. There are also different forms of selective microautophagy, such as micropexophagy (targeting peroxisomes), piecemeal microautophagy of the nucleus (targeting pieces of the nucleus), and micromitophagy (targeting mitochondria) [124].

## 2. Disordered Side of Self-Eating

A comprehensive bioinformatics analysis revealed that the majority of key human autophagy proteins contain IDRs [151]. This important observation suggests that intrinsic disorder plays an important role in regulating autophagy. A subsequent bioinformatics analysis of 95 autophagy-related proteins from the Human Autophagy Database (HADb) revealed that a large majority of these proteins are largely unstructured. In fact, 80.21% of these proteins contain at least one IDR longer than 30 amino acids, while 65.97% contain regions exceeding 50 amino acids [152]. This pivotal insight indicates that because IDRs do not fold into unique 3D structures, they give autophagy proteins the flexibility to interact with multiple partners simultaneously. This “shape-shifting” ability is likely what allows the autophagy machinery to assemble and disassemble so rapidly in response to cellular stress. It also suggests that many of these interactions might be regulated by PTMs (such as phosphorylation) that “switch” the IDR function on or off, as illustrated by the roles of PTMs in the regulation of ATG8 functions and the autophagy process [153].

In line with these earlier observations, sections below provide an overview of the functional intrinsic disorder in human proteins associated with macroautophagy, microautophagy, and their major interactors. These analyses indicate that the human autophagyome and microautophagyome, as well as their interactors, are profoundly enriched in intrinsic disorder. This structural plasticity allows autophagy-related proteins to engage in highly dynamic, multi-partner interactions, balancing high specificity with low affinity. Intrinsic disorder provides the conformational freedom necessary for these proteins to recognize diverse cargo, respond rapidly to metabolic stress, and facilitate the assembly of transient macromolecular complexes. We also present information on the LLPS potential of the autophagyome and microautophagyome members to indicate that beyond individual structural dynamics, LLPS acts as a major organizing principle in cellular degradation pathways. We categorize the identified autophagyome members based on their biophysical roles within biomolecular condensates. Specifically, we differentiate between “droplet drivers,” proteins that autonomously initiate phase separation to form templates for autophagosome biogenesis, and “droplet clients,” which partition into these compartments to undergo targeted degradation or to regulate enzymatic activity. Together, these insights underscore a sophisticated regulatory framework where intrinsic disorder and phase separation operate in tandem to orchestrate spatial and temporal control over macro- and microautophagy.

### 2.1. Macroautophagy

**Figure 1:**
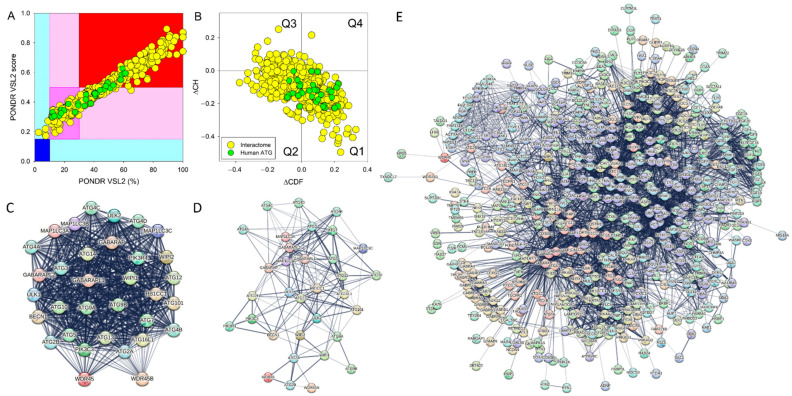
Global disorder and interactability analyses of major human autophagy-related proteins (green circles) and their interactors (yellow circles). (**A**). The PONDR^®^ VSL2 score vs. PONDR^®^ VSL2 (%) plot. Here, each point corresponds to a query protein, the coordinates of which are evaluated from the corresponding PONDR^®^ VSL2 data as its ADS and PPIDR. Color blocks are used to visualize proteins based on the accepted classification, with red, pink/light pink, and blue/light blue regions containing highly disordered, moderately disordered, and ordered proteins, respectively. Dark blue or pink regions correspond to the regions where PPIDR agrees with ADS, whereas areas in which only one of these criteria applies are shown by light blue or light pink (these are areas containing “mostly ordered plus” and “moderately disordered plus” proteins, see text). (**B**). Charge-Hydropathy and Cumulative Distribution Function (CH-CDF) analysis of query proteins. The ΔCH-ΔCDF plot is a two-dimensional representation that integrates both the CH plot, which correlates a protein’s net charge and hydrophobicity with its structural order [2,159], and the CDF, which cumulates disorder predictions from the N terminus to the C terminus of a protein, offering insight into the distribution of disorder residues [159]. The Y-axis (ΔCH) represents the protein’s distance from the CH boundary, indicating the balance between charge and hydrophobicity, while the X-axis (ΔCDF) represents the deviation of a protein’s disorder frequency from the CDF boundary [160]. Proteins are then stratified into four quadrants: quadrant 1 (bottom right) indicates proteins likely to be structured; quadrant 2 (bottom left) includes proteins that may be in a molten globule state or lack a unique 3D structure; quadrant 3 (top left) consists of proteins predicted to be highly disordered; quadrant 4 (top right) captures proteins that present a mixed prediction of being disordered according to CH but ordered according to CDF [160]. (**C**). Internal protein-protein interactome of the humane core autophagyome. This PPI network was generated using the STRING platform [161] using the confidence of 0.425 for the minimum required interaction score. This full STRING network contains the edges that indicate both functional and physical protein associations, and the line thickness indicates the strength of data support. This network contains 33 nodes and 516 edges, being characterized by the average node degree of 31.3 and an extremely high average local clustering coefficient of 0.982, which represents a nearly complete graph (clique). Access to the interactive version of this PPI network can be found on the STRING webpage via the following permalink URL: https://version-12-0.string-db.org/cgi/network?networkId=bZkPRbjhrZVJ (accessed on 2 July 2026). (**D**). Physical subnetwork generated by STRING using the confidence of 0.425 for minimum required interaction score. Here, the edges point out that the proteins are part of a physical complex. There are 33 nodes and 170 edges in this network, indicating that its average node degree and average local clustering coefficients are 10.3 and 0.632, respectively. Access to the interactive version of this PPI network can be found on the STRING webpage via the following permalink URL: https://version-12-0.string-db.org/cgi/network?networkId=bSp2WpPaUCAr (accessed on 2 July 2026). (**E**). PPI network centered at the core human autophagyome, highlighting shared interactors. This physical subnetwork was generated by STRING using the confidence of 0.425 for the minimum required interaction score. There are 527 nodes and 4449 edges in this network, which is characterized by the node degree of 16.9 and the average local clustering coefficient of 0.556. The interactive version of this network is available at the following permalink: https://version-12-0.string-db.org/cgi/network?networkId=bTEN8t9vHTmZ (accessed on 2 July 2026).

Table 1 and Figure 1A show that all 33 major autophagy-related proteins in humans are expected to contain noticeable levels of intrinsic disorder. The predicted percentage of intrinsically disordered residues (PPIDR) can be used for categorization of the query proteins as mostly ordered, moderately disordered, or, highly disordered if they are characterized by a PPIDR < 10%, 10% ≤ PPIDR < 30%, and PPIDR ≥ 30%, respectively [154,155]. Proteins can also be classified using their average disorder score (ADS) calculated by averaging the per-residue prediction scores across the entire sequence. Based on this criterion, the query proteins are considered ordered, moderately disordered, or highly disordered if their ADS values are ADS < 0.15, 0.15 ≤ ADS < 0.5, and ADS ≥ 0.5, respectively [154].

Although PPIDR and ADS are both metrics derived from the per-residue protein disorder predictors, they measure different aspects of structural disorder and are not identical, despite being correlated. For example, for a protein with the PPIDR of 100%, the ADS value can be anything in the range from 0.5 to 1.0, and vice versa, a protein with the PPIDR of 0% can have an ADS ranging from 0.0 to 0.5. Therefore, PPIDR acts as a “breadth” measure of how much of the sequence is affected, while ADS acts as a “depth” measure of the overall intensity of that disorder. In other words, PPIDR and ADS are distinct, complementary metrics of protein structure and should be used in combination to provide more robust classification of proteins as highly disordered (ADS ≥ 0.5, PPIDR ≥ 30%), moderately disordered (0.15 ≤ ADS < 0.5, 10% ≤ PPIDR < 30%) and mostly ordered (ADS < 0.15, PPIDR < 10%). This joint analysis also offers a means for the additional grouping, since the ADS and PPIDR outputs can disagree. In fact, some proteins are characterized by a PPIDR < 10% but have an ADS ≥ 0.15, whereas other proteins have 0.15 ≤ ADS < 0.5, but their PPIDR exceeds the 30% threshold. This defines two additional categories, “mostly ordered plus” and “moderately disordered plus”. The “plus” categories are particularly interesting, as they capture those edge cases, where a protein might have a few extremely disordered regions (boosting the ADS) or many slightly unstable regions (boosting the PPIDR).

Based on these criteria, 39.39%, 36.36%, and 24.24% of human main autophagy-related proteins (constituting the core autophagyome) are expected to be moderately disordered, “moderately disordered plus”, and highly disordered, respectively. This distribution is quite different from that of the human proteome (see Table 2), indicating that the core autophagyome relies more on the proteins from the “moderately disordered plus” category.

Figure 1B and Table 2 show results of the evaluation of the disorder status of a core autophagyome in terms of the ΔCH-ΔCDF plot. Here, the outputs of the CH and CDF analyses for query proteins are presented as a two-dimensional plot, where the Y-axis (ΔCH) measures distance from the charge-hydropathy boundary, while the X-axis (ΔCDF) measures deviation from the cumulative distribution function boundary. The plot employs four quadrants to categorize proteins: Q1 (bottom right) corresponds to the mostly ordered proteins (predicted ordered by both tools); Q2 (bottom left) shows hybrid/native molten globules (compact by CH, disordered by CDF); Q3 (top left) contains native coils/pre-molten globules (predicted disordered by both tools); and Q4 (top right) includes proteins predicted to be disordered by CH, but ordered by CDF. This analysis indicated that in comparison with the human proteome, the members of the autophagyome are more abundantly present in quadrants Q1 and Q2, whereas no members of the core autophagyome are present in quadrants Q3 and Q4 (see Figure 1B and Table 2).

The idea that the prevalent intrinsic disorder in autophagy-related proteins plays a functional role is supported by Table 1 and Figure 1C–E showing that these proteins are characterized by high interactability. In fact, among other parameters, Table 1 represents the p_LLPS_ values predicted for the core autophagyome members by FuzDrop and shows that more than two-thirds of this set correspond to proteins associated with LLPS. In fact, 10 and 15 autophagyome members are predicted to serve as droplet drivers (i.e., proteins with p_LLPS_ ≥ 0.60 capable of spontaneous LLPS) and droplet clients (i.e., proteins that have p_LLPS_ < 0.60 but contain droplet-promoting regions [DPRs], which can induce their partitioning into condensates). Curiously, a very noticeable fraction of the core autophagyome members (~24%) is not predicted to be related to LLPS, indicating that nearly a quarter of these core components may function independently of the phase-separation mechanism.

Figure 1C represents an internal PPI network within the core autophagyome and shows that both the number of edges and the average node degree of this network are close to the corresponding maximal possible values for a network with 33 edges (528 and 32, respectively). The extremely high local clustering coefficient (0.982) is expected in such a dense structure, where almost every neighbor of a node is also connected to every other neighbor. Because the expected number of edges for a random set of proteins of the same size and degree distribution drawn from the genome is 6, the core autophagyome has significantly more interactions among themselves than what would be expected (PPI enrichment *p*-value is <10^−16^), indicating that the proteins are at least partially biologically connected, as a group. Consideration of just the physical subnetwork representing complex formation revealed that all these proteins are engaged in at least 10 physical interactions with other members (see Figure 1D), which is characteristic of highly dense protein complexes, where each subunit acts as a hub that interacts with multiple other components within the same complex.

Figure 1E represents a total physical PPI network centered at the human core autophagyome and shows that this network is characterized by high connectivity as well. To generate this network, the custom value of 0.425 was used for the minimum required interaction score, and the number of interactors in the first shell was set to 500 (the maximal permitted number of interactors in STRING). The minimum required interaction score of 0.425 is above the standard 0.400 medium-confidence threshold in STRING. This ensures that only interactions with strong evidence are included, filtering out low-confidence, noisy connections. Setting the first shell to 500 nodes (the maximum allowed) maximizes the network size, providing a comprehensive overview of the functional neighborhood of query proteins. Because the resulting network contains 527 proteins, the used settings for the group of 33 core autophagyome members allowed the generation of a network containing close to the maximally available number of nodes (533 = 33 core + 500 interactors). The high yield of 527 nodes suggests that the core autophagyome functions as a tightly knit module within the wider human protein interactome, rather than as isolated components. The autophagyome-centered interactome exhibits a strong community structure and high interconnectedness among neighbors, being characterized by a high clustering coefficient of 0.556, and thereby fitting a small-world network model, where neighbor nodes of a particular protein have a high probability of interacting with each other. This dense, organized structure is tailored for fast and efficient information flow, allowing the autophagyome to respond quickly to metabolic or environmental signals.

Figure 1A,B and Table 2 show that the interactors of the core autophagyome are characterized by high intrinsic disorder levels as well, with the vast majority of them being classified as moderately disordered (28.84%), “moderately disordered plus” (32.45%), and highly disordered (35.86%) proteins. This set does not have any mostly ordered proteins and contains only 0.95% of “mostly ordered plus” proteins. With over 99% of these interactors falling into the disordered categories, it is clear that the core autophagy machinery requires significant structural flexibility. This lack of a rigid shape likely allows these proteins to quickly adapt, bind to multiple partners, and facilitate the rapid signaling changes needed during cellular stress or recycling.

A few illustrative examples of the functional utilization of intrinsic disorder by the core autophagyome members come from studies in yeast. It was shown that *Saccharomyces cerevisiae* Atg13 (UniProt ID: Q06628; PPIDR_PONDR VSL2_ = 71.54%, p_LLPS_ = 0.9929, a homolog of human ATG13) has a long C-terminal IDR (residues 272–738), which is highly phosphorylated by Tor kinase complex 1 (TORC1) under nutrient-rich conditions but is dephosphorylated in response to starvation [162] and interacts with two distinct Atg17 molecules (UniProt ID: Q06410; PPIDR_PONDR VSL2_ = 23.74%, p_LLPS_ = 0.1256) using two binding regions (residues 359–389 and 424–436) located within this IDR [163]. Under starvation conditions, this protein also interacts with Atg1 (UniProt ID: P53104; PPIDR_PONDR VSL2_ = 42.81%, p_LLPS_ = 0.5688, which is the most upstream factor in autophagy that forms a complex to initiate autophagosome formation during cellular stress) using another segment of the C-terminal IDR (residues 460–521) [163,164]. Therefore, Atg13 uses an intrinsically disordered C-terminal region to tether Atg1 and the Atg17-Atg31-Atg29 subcomplex leading to the formation of the minimal unit of this complex [163]. Importantly, all the other proteins involved in the assembly of the Atg1 complex and recruitment of Atg9-containing vesicles are predicted to be highly disordered and show high LLPS potential: Atg9 (UniProt ID: Q12142; PPIDR_PONDR VSL2_ = 46.54%, p_LLPS_ = 0.5341); Atg29 (UniProt ID: Q12092; PPIDR_PONDR VSL2_ = 61.97%, p_LLPS_ = 0.7611); Atg31 (UniProt ID: Q12421; PPIDR_PONDR VSL2_ = 62.24%, p_LLPS_ = 0.7176). These observations suggest that the long IDRs and high LLPS potential are crucial for facilitation of Atg1 complex assembly and Atg9-containing vesicle recruitment [165], ensuring the spatiotemporal coordination required for autophagosome biogenesis [165,166]. We will discuss the LLPS of the Atg1 complex further in the third chapter.

The disordered N- and C-terminal regions (NTR and CTR, respectively) of yeast Atg9, which is the only transmembrane protein in the core autophagy machinery, acting as a lipid transporter with a highly conserved membrane-bound core, mediate its interaction with other autophagy factors. For example, the short disordered PLF motifs within the Atg9-NTR are used to bind Atg11, facilitating the recruitment of Atg9-containing vesicles to the cargo [167]. Yeast Atg1, Atg9, Atg13, and Atg17 are orthologs of human autophagy-related proteins ULK1/ULK2, ATG9A, ATG13, and RB1CC1/FIP200, respectively, all of which are predicted to have high disorder levels (see Table 1). In human cells, there are no direct sequence orthologs for the yeast autophagy proteins Atg29 and Atg31; however, human ATG101, which does not share high sequence similarity with Atg29 or Atg31, structurally resembles and functionally fulfills a similar role in binding to ATG13 and RB1CC1 to stabilize the complex, much like Atg31-Atg29 does in yeast [168]. Therefore, these observations indicate that the disorder-based functionality of the core autophagyome is conserved from yeast to human.

Another core autophagyome member, the Atg12 protein, is indispensable for the highly conserved ubiquitin-like conjugation system operating in the macroautophagy/autophagy pathway [169]. In yeast autophagy, the ubiquitin-like protein Atg12 (UniProt ID: P38316; PPIDR_PONDR VSL2_ = 66.67%, p_LLPS_ = 0.8704) is covalently linked to Atg5 (UniProt ID: Q12380; PPIDR_PONDR VSL2_ = 15.31%, p_LLPS_ = 0.1085) via the action of a ubiquitin-like conjugating system involving Atg7 (UniProt ID: P38862; PPIDR_PONDR VSL2_ = 13.33%, p_LLPS_ = 0.1085) as an E1-like activating enzyme and Atg10 (UniProt ID: Q07879; PPIDR_PONDR VSL2_ = 10.18%, p_LLPS_ = 0.1349) as an E2-like conjugating enzyme. The resulting Atg12–Atg5 conjugate subsequently binds Atg16 (UniProt ID: Q03818; PPIDR_PONDR VSL2_ = 80.67%, p_LLPS_ = 0.1989) to form an E3-like ligase complex. This complex facilitates the separate conjugation of Atg8 to phosphatidylethanolamine (PE) during autophagosome formation [169]. It was shown that the NTR of the yeast Atg12 (residues 1–100) is intrinsically disordered and is crucial for the functionality of this protein [169]. Bioinformatics analyses indicate that, on the one hand, the intrinsic disorder propensities of human autophagy-related proteins, ATG7, ATG3, and members of the ATG12–ATG5-ATG16L1 complex, are comparable to their yeast orthologs (see Table 1), suggesting that the functional role of IDRs in the conjugation system is evolutionarily conserved. On the other hand, the low LLPS potential of many members of the autophagy-related conjugation system indicates that these components likely function to promote the transition from a liquid-like assembly to a more stable, membrane-bound structure. Unlike earlier acting complexes (such as the Atg1/ULK complex) that utilize high LLPS potential for condensate formation, the Atg8 and Atg12 conjugation machinery operates in part to convert phagophores, autophagosome precursors, into closed, mature autophagosomes.

### 2.2. Microautophagy

As noted above, both macroautophagy and microautophagy separate autophagic cargo from the remaining cytoplasm via membranes; a key difference is that the former involves the formation of autophagosomes, whereas the latter utilizes direct uptake at the limiting membrane of the degradative organelle. Microautophagy is controlled by two major sets of proteins, the ESCRT machinery, which facilitates membrane deformation, fission (scission), and cargo sequestration, and some core ATG proteins, which, in some types of selective microautophagy (such as pexophagy and nucleophagy in yeast), are required to form a phagophore-like structure known as the Micropexophagic Membrane Apparatus (MIPA) [129,170]. In human cells, the ESCRT-0, ESCRT-I, and ESCRT-III complexes function as the primary regulators of fission-type microautophagy. They mediate the necessary membrane remodeling, specifically the invagination and scission of the endolysosomal surface, to engulf and degrade cytoplasmic cargo [171,172,173]. Several human core ATG proteins (such as ATG5, ATG7, ATG12, ATG14, BECN1, GABARAP, LC3, and PIK3C3/VPS34) are involved in fusion-type microautophagy [170]. Furthermore, soluble N-ethylmaleimide-sensitive factor attachment protein receptor (SNARE) proteins are involved in microautophagy as well, as they mediate the direct fusion between the lysosomal membrane and the cargo or sequestering vesicles [123,129]. A detailed description of the roles of the ESCRT machinery, as well as ATG proteins and SNAREs in different types of microautophagy is outside the scope of this study, and interested readers can find related information in dedicated reviews.

Some intrinsic-disorder-based features of the major microautophagy-related proteins are listed in Table 3, which shows that the vast majority of these proteins (37 of 51 or 72.5%) are predicted as highly disordered. Furthermore, 8 proteins (15.7%) belong to the “moderately disordered plus” category, with the remaining 6 proteins (11.8%) being moderately disordered. This makes the microautophagy-related proteins extraordinarily flexible and dynamic, indicating that structural flexibility and the lack of a fixed 3D structure are likely fundamental to how these proteins function during microautophagy.

**Figure 2:**
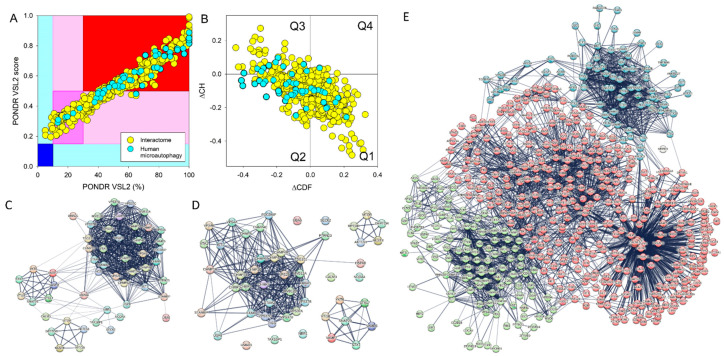
Global disorder and interactability analyses of major human microautophagy-related proteins (cyan circles) and their interactors (yellow circles). (**A**). The PONDR^®^ VSL2 score vs. PONDR^®^ VSL2 (%) plot. (**B**). The ΔCH-ΔCDF analysis of query proteins. (**C**). Internal protein-protein interactome of the human microautophagyome. This PPI network was generated using the STRING platform using the confidence of 0.425 for minimum required interaction score. This full STRING network contains the edges that indicate both functional and physical protein associations, and the line thickness indicates the strength of data support. This network contains 51 nodes and 513 edges, being characterized by the average node degree of 20.1 and a high average local clustering coefficient of 0.853. Access to the interactive version of this PPI network can be found on the STRING webpage via the following permalink: https://version-12-0.string-db.org/cgi/network?networkId=bQEsE3jDdhA9 (accessed on 2 July 2026). (**D**). Physical subnetwork generated by STRING using the confidence of 0.625 for minimum required interaction score. Here, the edges point out that the proteins are part of a physical complex. There are 51 nodes and 315 edges in this network, indicating that its average node degree and average local clustering coefficients are 12.4 and 0.738, respectively. Access to the interactive version of this PPI network can be found on the STRING webpage via the following permalink: https://version-12-0.string-db.org/cgi/network?networkId=bhrJSr7CJ8mO (accessed on 2 July 2026). (**E**). PPI network centered at the human microautophagyome representing shared interactors. This physical subnetwork was generated by STRING using the confidence of 0.625 for minimum required interaction score. There are 546 nodes and 3553 edges in this network, which is characterized by the node degree of 13 and the average local clustering coefficient of 0.648. The network is clustered using the k-means clustering algorithm of STRING that find a defined number of clusters based on their centroids. The three functional clusters are: 358 “red” proteins involved in regulation of localization, enzyme binding, and neuron projection; 110 “green” proteins related to multivesicular body assembly and ESCRT; and 77 “blue” proteins involved in organelle membrane fusion and the SNARE complex. The interactive version of this network is available at the following permalink: https://version-12-0.string-db.org/cgi/network?networkId=blP0ZBlPUM5j (accessed on 2 July 2026).

Figure 2 and Table 1 and Table 2 provide further support to the prevalence of IDPs and IDRs in microautophagy, suggesting that intrinsic disorder plays a key, yet previously mostly underappreciated, role in the regulation of the microautophagy pathways. With almost two/thirds of its proteins being highly disordered (see Figure 2 and Table 1 and Table 2), microautophagy represents a standout example of a cellular process powered by intrinsic disorder. While macroautophagy uses a “delivery truck” (the autophagosome) to transport cargo, microautophagy is more direct: the lysosome or vacuole simply folds its own membrane around nearby material to “eat” it. To handle this constant membrane reshaping and grab various targets on the fly, the process relies on these flexible, unstructured proteins rather than rigid ones. Therefore, IDRs in the microautophagy-related proteins are likely to be essential for the functional adaptability of the microautophagy machinery, enabling dynamic, low-affinity, and high-specificity interactions with multiple partners during cargo recognition, invagination, protrusion, or septation of the lysosomal or vacuolar membrane, and formation of single-membrane vesicles.

Members of the microautophagyome form a tightly-linked internal PPI network as evidenced by Figure 2C. This important observation suggests that microautophagy represents a highly integrated functional unit, despite the diverse origins of its components. In fact, microautophagy relies on the physical and functional interactions among the proteins from three distinct systems: ESCRT that drives membrane remodeling and scission events, MTORC1 that acts as a master regulator of catabolic pathways, and SNAREs that mediate the fusion of autophagosomal membranes with lysosomes. This tightly-linked internal PPI network highlights that these machineries do not operate in isolation. Instead, they form a coordinated complex where metabolic signals (MTORC1) directly modulate the physical sorting machinery (ESCRT) to execute selective degradation. This cross-talk is surprising as these systems were historically studied as independent regulators of endocytosis, signaling, and membrane fusion, respectively. This idea is illustrated by Figure 2D representing a physical subnetwork of the microautophagyome and showing three disjoint clusters that maps specific molecular machineries involved in these distinct yet converging processes.

A total physical PPI network centered at the human microautophagyome is shown in Figure 2E. This network was generated using the minimum required interaction score of 0.625 and 500 interactors in the first shell. These settings yielded a network with 546 edges (close to the maximally available number of nodes of 551). In line with the presence of three different microautophagy-related machineries (ESCRT, MTORC1, and SNAREs) the use of a k-means clustering algorithm in the STRING database partitioned the microautophagyome-centered PPI network into three distinct groups of functionally related proteins. Figure 2A,B and Table 2 show that the interactors of the microautophagyome are also predicted to contain high levels of intrinsic disorder. In fact, the vast majority of the microautophagyome interactors are moderately disordered (28.94%), “moderately disordered plus” (27.66%), and highly disordered (42.12%) proteins. Therefore, not only the microautophagyome but its interactome as well are noticeably more disordered than the corresponding macroautophagy-related proteins.

Finally, Table 3 represents the FuzDrop-generated p_LLPS_ values for the microauto-phagyome and shows almost all these proteins are associated with LLPS. In fact, only three of these proteins (5.9%) were predicted to be unrelated to LLPS (being characterized by very low p_LLPS_ values and containing DPRs), whereas 27 (52.9%) and 21 (41.2%) microautophagy proteins were predicted as droplet drivers and droplet clients, respectively. Therefore, nearly all (~94%) microautophagy-related proteins have the potential to form or interact with liquid-like droplets. These observations highlight a strong, pervasive link between the microautophagy machinery and LLPS, with microautophagy being heavily driven by, or organized through, the formation of liquid-like droplets. This suggests that the microautophagyome is remarkably “phase-separation friendly,” being highly optimized to utilize phase separation for its function, organization, and regulation. The high percentage of droplet drivers (52.9%) among the microautophagy machinery itself is responsible for forming the scaffolds that facilitate cargo sequestration.

## 3. Mechanisms of Autophagy-Related Proteins That Undergo LLPS in the Regulation of Autophagy

**Figure 3:**
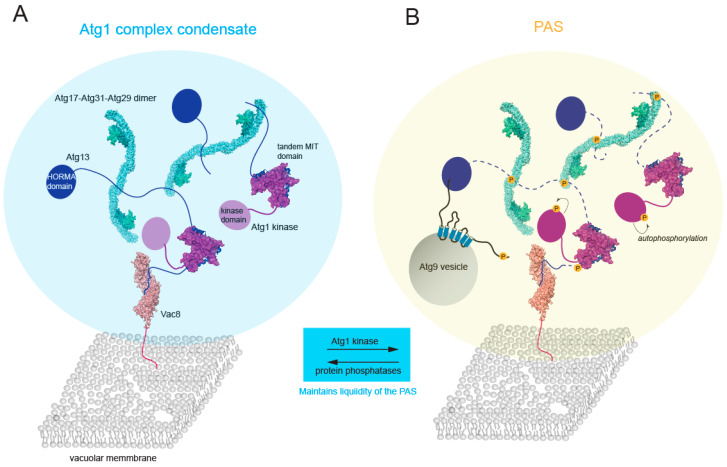
Atg13 drives LLPS of the Atg1 complex and formation of the PAS. (**A**) Under nutrient-rich conditions, the hyperphosphorylation of Atg13 by TORC1 prevents assembly of the Atg1 complex. Stress conditions inhibit activity of TORC1 and PP2C phosphatases dephosphorylate the Atg13 IDR on multiple sites (S379 in Atg17 LR, S428, S429 in Atg17BR, and S484, S494, S496, S515, and S517 in MIM(C) that is bound to the Atg1 tMIT domain (PDB ID: 4P1N). These modifications drive LLPS of the Atg1 complex (comprising the Atg17-Atg31-Atg29 complex (PDB ID: 4HPQ), Atg1 kinase, and Atg13) near the vacuolar membrane through the Atg13 IDR-Vac8 interaction (PDB ID: 6KBM)). (**B**) The phase separation activates Atg1 via autophosphorylation, and the kinase phosphorylates Atg13 and Atg9. This process recruits downstream factors, including Atg9-containing vesicles and, thereby, forming the PAS near the vacuole. The phosphorylation of Atg13 by Atg1 releases some interactions within the PAS followed by dephosphorylation of Atg13 by protein phosphatases. This periodic phosphorylation and dephosphorylation of Atg13 keeps the PAS in a liquid state. Dashed lines in phosphorylated Atg13 depict released interactions.

For autophagy initiation in yeast, it is Atg13 that drives phase separation of the Atg1 kinase complex, a prerequisite for formation of the phagophore assembly site (PAS) [174]. As mentioned above, under nutrient-rich conditions, Atg13 is hyperphosphorylated by TORC1, which keeps the components of the Atg1 complex apart. Stress conditions, such a starvation, inhibit TORC1 activity, and Atg13 is dephosphorylated by PP2C phosphatases. This modification on serine residues in the Atg13 IDR triggers multivalent interactions of Atg13 with the Atg1 kinase and the Atg17 scaffolding protein [163,164]. Thereby, the Atg13 IDR drives LLPS of the Atg1 complex (Figure 3). Vac8, a protein attached to the vacuolar membrane, binds the C-terminal region of Atg13. The Atg13-Vac8 dimer maintains the phase-separated Atg1 complex near the vacuolar membrane. It remains to be determined whether formation of the dimer is also regulated by phosphorylation. The phase-separated Atg1 complex triggers activation of Atg1 via autophosphorylation. The active kinase subsequently phosphorylates Atg9 and Atg13, which has two important outcomes. The first outcome is the recruitment of downstream factors and formation of the PAS near the vacuole (Figure 3). The second outcome is the breakage of some interactions of phosphorylated Atg13 with Atg1 and Atg17, which allows for protein mobility and dephosphorylation of Atg13 by phosphatases. The dephosphorylation makes Atg13 prone to re-establishing the Atg1 complex. These periodic phosphorylation-dephosphorylation cycles in the Atg13 IDR are assumed to maintain the PAS in a liquid state [174,175].

**Figure 4:**
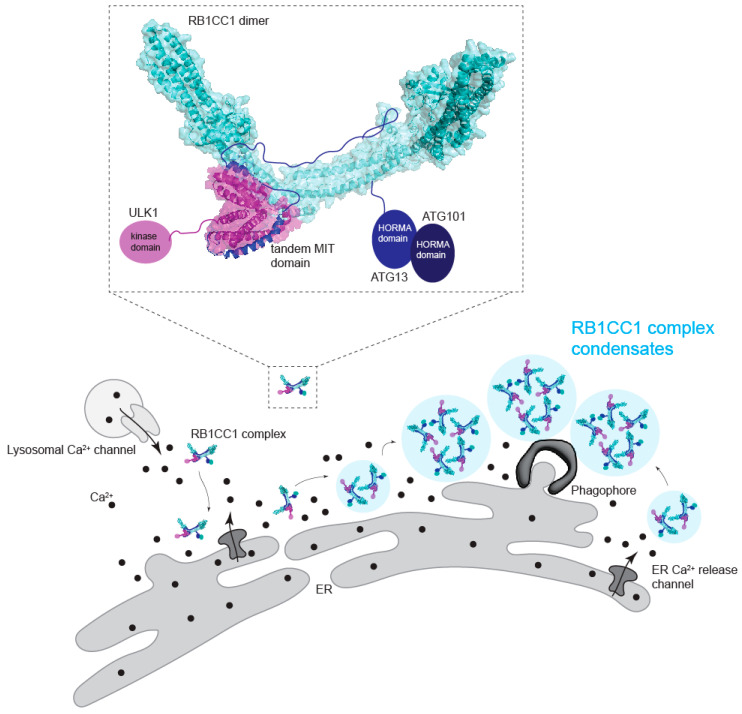
The RB1CC1 complex phase separates near the ER in response to a Ca^2+^ gradient. Conditions that induce autophagy stimulate accumulation of Ca^2+^ on the outer surface of the ER. This process induces LLPS of the RB1CC1 (PDB ID: 8SOI). The RB1CC1 condensates transfer to a site of phagophore origination.

In more complex eukaryotes, the autophagy initiation complex comprises the ULK1 kinase, RB1CC1/FIP200, ATG13, and ATG101 subunits. The RB1CC1 scaffolding protein undergoes LLPS in response to Ca^2+^ gradients on the outer membrane of the ER. Ca^2+^ is released from the lysosome and the ER upon autophagy induction. The phase-separated RB1CC1 condensates regulated by ATG9-containing vesicles travel along the ER surface to a site of phagophore initiation [176] (Figure 4).

**Figure 5:**
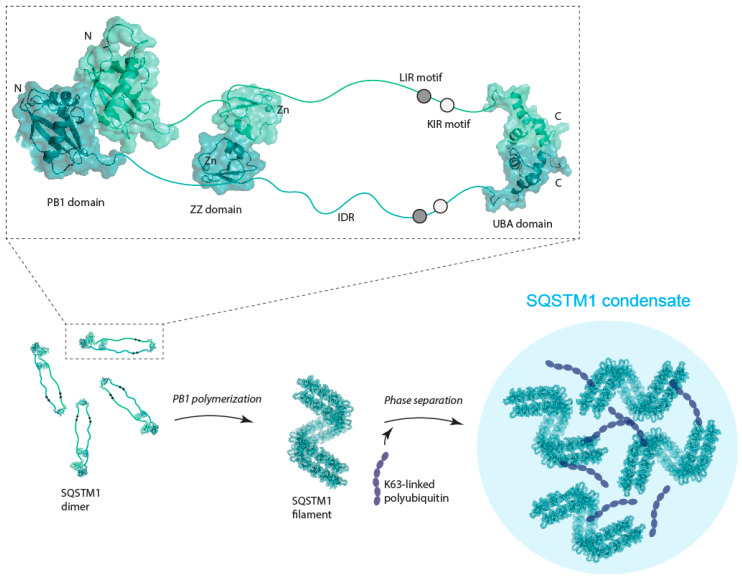
SQSTM1 filaments bound to ubiquitin drive LLPS. The PB1, ZZ (PDB ID: 5YP7), and UBA (PDB ID: 2KNV) domains mediate the dimerization of SQSTM1, and polymerization of the PB1 domain forms SQSTM1 filaments (PDB ID: 9HGE) of a helical shape. The interaction between these filaments and K63-linked polyubiquitin chains drives the LLPS of SQSTM1.

In selective autophagy, the specific cargo is delivered to the degradative organelle (lysosome or vacuole) via autophagy receptors. The receptors connect the cargo with the Atg8-family proteins, conjugated to PE on the growing phagophore, by employing a specific short linear motif (SLiM) that fits hydrophobic pockets on the surface of ATG8s. This SLiM, termed AIM (Atg8-interacting motif in yeast) or LIR (LC3-interacting region in more complex eukaryotes), resides in IDRs of the receptors [177]. One of the best characterized receptors for ubiquitinated cargo destined for degradation in the lysosome is SQSTM1/p62. The SQSTM1 protein comprises three folded dimerizing domains, Phox and Bem1 (PB1), zinc finger (ZZ), and ubiquitin-associated (UBA), linked by two IDRs, where the longer one carries the LIR and KEAP1-interacting region (KIR) motifs (Figure 5). The PB1 domain is responsible for the polymerization of SQSTM1 into filaments [178] and the UBA domain for binding polyubiquitin chains that control the length of the SQSTM1 filaments and their sequestering activity [179,180]. The K63 ubiquitin chains are essential for the phase separation of SQSTM1 into droplets [181] (Figure 5). LLPS of SQSTM1 is facilitated not only by its LIR motif but also by NBR1, an autophagic receptor comprised of PB1, ZZ, and UBA domains, as well as two LIRs [182]. When SQSTM1 separates into gel-like condensates and interacts with ATG8s, it does not become mere cargo, but it actively functions as a scaffold for autophagosome formation [183]. Inner fluidity of the condensates is essential for the SQSTM1 function in selective autophagy because amyotrophic lateral sclerosis (ALS)-related and frontotemporal degeneration (FTD)-related mutations in the LIR and KIR of SQSTM1 exhibit a decreased droplet fluidity [184]. SQSTM1 is a versatile receptor for both proteins and lipids. A recent study showed that SQSTM1 filaments encapsulate lipid droplets, and Ca^2+^ released from the ER assists in layering of the filaments [178]. Next to ions, non-autophagic proteins can control the LLPS of SQSTM1. For example, myosin 1D (MYO1D), a branched actin-associated motor protein drives LLPS of SQSTM1 [185].

**Figure 6:**
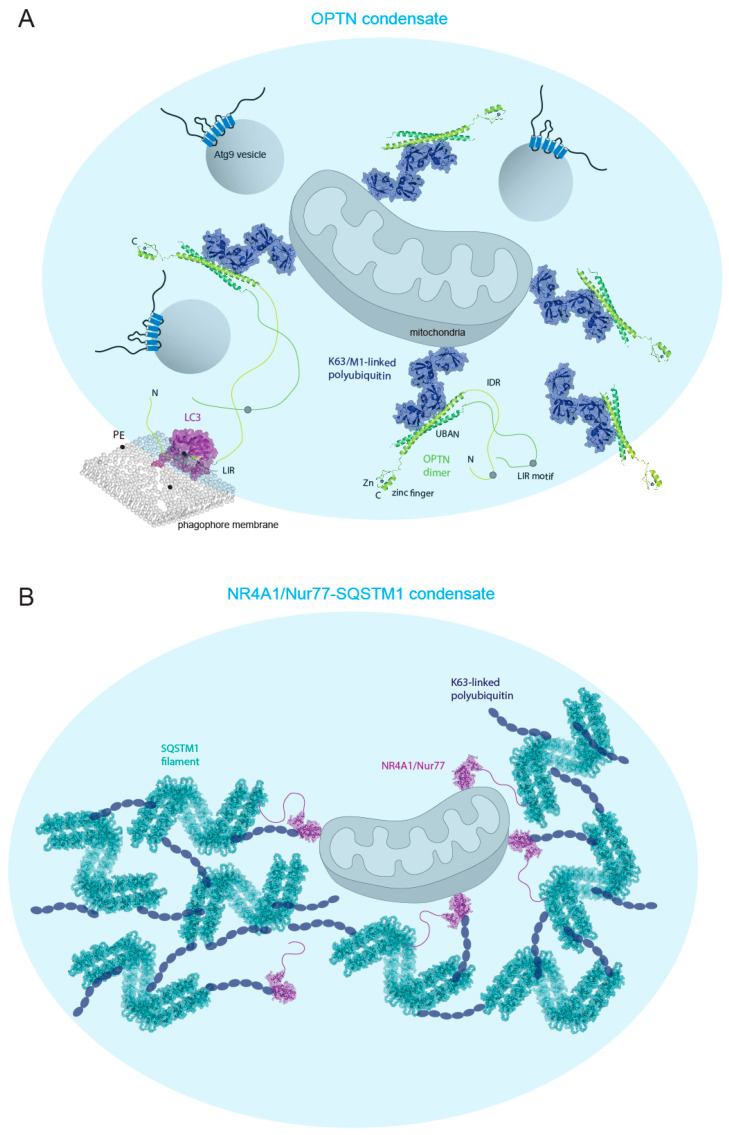
Phase separation in mitophagy. (**A**) LLPS of OPTN recruits Atg9-containing vesicles into a liquid condensate during mitophagy. The K63-linked ubiquitin chain bound to the UBAN domain of OPTN (PDB ID: 5B83) upstream of the zinc finger domain (PDB ID: 2LO4) attaches to mitochondria. The LIR motif in OPTN bound to LC3 on the phagophore membrane (PDB ID: 2LUE) ensures engulfment of the condensate with the mitochondria by the growing phagophore. Folding of the OPTN IDR into two N-terminal coiled-coils flanking the LIR motif is not depicted. (**B**) The nuclear receptor NR4A1 drives mitophagy by promoting liquidity of SQSTM1 droplets. The ligand binding domain (LBD) of NR4A1 binds to mitochondria and the IDR is essential for maintaining the liquidity of the phase-separated condensates. In the absence of the NR4A1 IDR, damaged mitochondria are sequestered, but SQSTM1 does not bind to LC3 via its LIR motif. NR4A1 is represented based on AlphaFold3 model. Polymerization of the PB1 domain in SQSTM1 forms filaments (PDB ID: 9HGE) of a helical shape.

Optineurin (OPTN) is another important autophagy receptor. This protein is mostly intrinsically disordered. It carries an LIR motif flanked by two coiled-coil domains at its N terminus, a dimerizing helical domain in the middle of the amino acid sequence, and a zinc finger domain at the C terminus. A recent study [186] showed that OPTN undergoes LLPS, and the OPTN condensate recruits ATG9-containing vesicles during mitophagy (Figure 6A). Both K63- and M1-linked polyubiquitin chains connect OPTN to damaged mitochondria, and the LIR motif in OPTN links the condensate to LC3–PE on the phagophore membrane (Figure 6A). The growing phagophore engulfs the OPTN condensate containing the damaged mitochondria for ultimate degradation in the autolysosome. An alternative pathway for the degradation of dysfunctional mitochondria is a mechanism involving NR4A1/Nur77. After the induction of mitophagy, an anti-inflammatory compound isolated from the medicinal herb *Tripterygium wilfordii*, calestrol, binds NR4A1, a nuclear transcription factor, and triggers its translocation to damaged mitochondria. The phase separation of NR4A1 promotes the liquidity of SQSTM1 condensates. Specifically (Figure 6B), NR4A1 utilizes its C-terminal ligand binding domain to attach to dysfunctional mitochondria. The IDR of NR4A1 binds the PB1 domain of SQSTM1 and promotes fluidity of the NR4A1-SQSTM1 condensate. In the absence of the NR4A1 IDR, the condensate is rigid, and the LIR motif in SQSTM1 cannot connect to LC3 [187], demonstrating that the liquidity of SQSTM1 droplets is a critical parameter.

**Figure 7:**
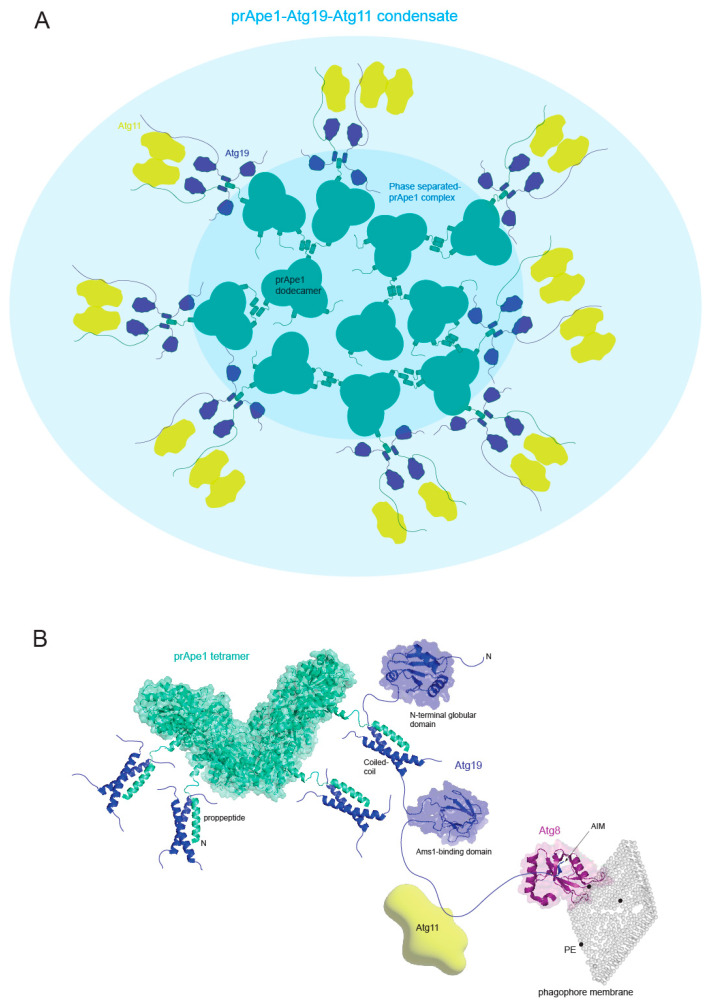
Phase separation in the selective Cvt pathway. (**A**) The prApe1 dodecamers phase separate into semi-liquid droplets via a trimeric coiled-coil structure formed by prApe1 propeptides. Atg19 prevents unlimited growth of the prApe1 droplet by replacing two of the prApe1 propeptides with its own coiled-coil domain in the trimer. The Atg19 molecules “float” on the surface of the phase separated prApe1 droplet, and the N-terminal globular domain of Atg19 is essential in the flotation process. This Atg19-prApe1 configuration keeps the proteins mobile in the droplet. The C-terminal IDR of Atg19 binds Atg11, which increases the local concentration of Atg11 and promotes its self-association via the second and third coiled-coil region and then phase separation. A low-affinity interaction between the receptor and the cargo allows for the receptor’s mobility and phase separation of Atg11. This assembly is the condensate inside the condensate. (**B**) Atg19 in specific interactions with its binding partners. Downstream of the Atg19 N-terminal globular domain (represented by the AlphaFold3 model), Atg19 binds prApe1 via a 3-helical bundle, where one helix is derived from the prApe1 propeptide and two helices are coiled-coil domains of the two receptor molecules (PDB ID: 5JGE). One prApe1 tetramer of the dodecamer is shown (PDB ID: 5JH9). Downstream of the Ams1-binding domain (PDB ID: 2KZB) of Atg19 is the C-terminal tail that binds the 4th coiled-coil domain of Atg11. The AIM motif at the very end of the Atg19 C terminus attaches on the surface of Atg8 (PDB ID: 2ZPN) conjugated to PE in the phagophore membrane.

A group of autophagy-related proteins that undergo LLPS operates in the biosynthetic cytoplasm-to-vacuole targeting (Cvt) pathway in yeast. This pathway ensures transport of alpha-mannosidase (Ams1) and precursor amiopeptidase I (prApe1), resident vacuolar hydrolases, to the vacuole. The prApe1 oligomerizes into dodecamers that expose propeptides on their surface. The propeptides form a trimeric coiled-coil structure that facilitates phase separation of the dodecamers into prApe1 droplets. Atg19 is an autophagy receptor that controls the size of the droplets by replacing two out of the three coiled-coil helices in the trimeric structure with its own coiled-coil helices (Figure 7A). Atg19 floats on the surface of phase-separated prApe1 dodecamers via the action of the Atg19 N-terminal globular domain (Figure 7B). Flotation is essential for maintaining mobility of the Atg19-prApe1 complex [188,189]. The C-terminal IDR of Atg19 binds the Atg11 scaffolding protein. An increase in the local concentration of Atg11 near prApe1 promotes the phase separation of Atg11 via the second and third coiled-coil domain. LLPS of Atg11 needs a low affinity binding between Atg19 and prApe1 [190], possibly due to accessibility of the Atg11 binding site in Atg19. The C-terminal AIM motif in Atg19 connects the prApe1-Atg19-Atg11 condensate to Atg8 conjugated to PE on the growing phagophore (Figure 7B).

## 4. Factors That Modulate the LLPS Potential of Autophagy Proteins and the Involvement of IDRs

### 4.1. Phosphorylation

Biomolecular condensates formed by LLPS rely on the self-association of scaffold proteins that display a high valency due to multiple interaction segments. Cells modulate the formation of liquid droplets by changing the concentration or solubility of scaffold proteins. IDRs are important components of scaffolding as they have a high content of charged amino acid residues that are engaged in the interactions with water molecules. PTMs such as phosphorylation or acetylation, taking place mostly in IDRs, can tune the composition of charges in IDRs and, thereby, decrease or increase the water solubility of scaffolds. The aforementioned phosphorylation-dephosphorylation cycling of the Atg13 IDR is a clear example of modulation of the PAS liquidity during autophagy initiation [174]. LLPS in selective autophagy also relies on PTMs, particularly on autophagy receptors that interact with the autophagy machinery and ubiquitinated cargo. RB1CC1/FIP200, a subunit of the autophagy initiation complex in more complex eukaryotes, binds the SQSTM1 condensates containing ubiquitinated cargo. The phosphorylation of SQSTM1 enhances the interaction of its RB1CC1/FIP200-interacting motif (FIR) motif, which overlaps with the LIR motif in its IDR [191,192], with the Claw domain of RB1CC1. This interaction slows down LLPS of SQSTM1 droplets. LC3B then outcompetes RB1CC1 from SQSTM1 via the LIR motif binding pockets to recruit the cargo to the phagophore [193]. Another phosphorylation of SQSTM1, on Ser403 by TBK1 in the UBA domain, enhances polyubiquitin chain binding and promotes LLPS of SQSTM1 [181,194]. SQSTM1 is also ubiquitinated on K420 by the KEAP1-CUL3 (cullin 3) complex. A KEAP1 homodimer binds to two KIR motifs in the IDRs of an SQSTM1 dimer and promotes higher-order oligomers [180]. Analogously to SQSTM1, the phosphorylation of OPTN by TBK1 promotes the formation of OPTN filaments [195] and facilitates polyubiquitin chain binding to OPTN [196,197]. Interestingly, the M1-linked polyubiquitin chains induce LLPS of OPTN more efficiently than the K48- and K63-linked polyubiquitin chains [195].

### 4.2. Acetylation

As mentioned above, RB1CC1 undergoes LLPS (Figure 4) [176]. The N-terminal IDR of RB1CC1 driving this process is acetylated by the acetyltransferase CREBBP on several lysine residues, where K276 is a major acetylation site. Because K276 is also a ubiquitination site, the acetylation of K276 suppresses ubiquitination and subsequently the ubiquitin-dependent degradation of RB1CC1. Cancer cells hijacking autophagy for their survival need the intact IDR with acetylated K276 for functional RB1CC1 in autophagy [198].

### 4.3. Binding of a Protein

Binding of a protein partner is another factor affecting the LLPS potential in auto-phagy. One example is the aforementioned Atg19-prApe1 interaction preventing unlimited growth of the prApe1 biocondensates. Another example is seen with transcription factor EB (TFEB), a master regulator of lysosomal biogenesis and autophagy that activates the formation of autophagosomes [199]. TFEB undergoes LLPS and this process is regulated by inositol polyphosphate multikinase (IPMK). IPMK does not act on LLPS via its kinase activity but operates rather via attaching its C-lobe domain to TFEB, which inhibits LLPS of TFEB [200]. Another example is the interaction between SMAD specific E3 ubiquitin protein ligase 1 (SMURF1) and SQSTM1. SMURF1 is an E3 ligase that binds to SQSTM1 and, thereby, increases its capacity to form liquid droplets [201]. Another E3 ligase, SYVN1/HRD1, regulates the LLPS potential of SNAP29. This SNAP protein undergoes LLPS [202] and forms a complex with syntaxin 17 (STX17) and VAMP8. SYVN1/HRD1 binds SNAP29 and suppresses its phase separation [202].

### 4.4. Modulation by SLiM

IDRs execute their functionality by engaging modules that are often evolutionarily conserved. These modules are either short linear motifs (SLiMs) or molecular recognition features (MoRFs) [203]. The LIR/AIM motif in ATG8/Atg8 proteins is a SLiM [177]. The LIR of SQSTM1 is essential for LLPS and condensate formation. Testing whether the LIR motif is required for SQSTM1 clustering in the presence of ubiquitin shows that cluster formation is substantially reduced in the LIR mutant relative to the wild type [182]. In addition, the LIR mutant also exhibits diminished recruitment to preformed SQSTM1-ubiquitin assemblies. The molecular basis for this observation remains to be elucidated.

## 5. Biocondensophagy—Autophagic Degradation of Biomolecular Condensates Formed via LLPS

Proteins associated with neurodegenerations often undergo LLPS before their pathological aggregation. MAPT/tau, a soluble intrinsically disordered protein, undergoes LLPS in a concentration-dependent manner. Phase-separating MAPT/tau is heavily hyperphosphorylated on multiple sites in the repeat domain, proline-rich region, and in the N-terminal insets. LLPS of phosphorylated MAPT/tau initiates protein aggregation and fibrillation [204,205,206]. A recent study showed that myricetin, a natural flavonoid found in fruits, vegetables, tea, and wine, slows down LLPS of MAPT/tau. This natural compound also inhibits the phosphorylation and pathological aggregation of MAPT/tau in cells. Mechanistically, myricetin inhibits MTOR and increases the levels of ATG5 and lipidated LC3B, which initiates autophagic clearance of phosphorylated MAPT/tau. In fact, myricetin stabilizes the interaction between MAPT/tau and ATG5 [207]. Interestingly, there is another strategy for the degradation of MAPT/tau via autophagy. This strategy bypasses the LLPS of MAPT/tau. Human cells have ring finger protein 216 (RNF216), an E3 ubiquitin ligase that operates as an autophagy receptor and facilitates removal of MAPT/tau. Specifically, RNF216 assembles K11- and K63-linked ubiquitin chains that promote LLPS of RNF216. MAPT/tau is ubiquitinated and accumulates in the RNF216 droplets. Sequestering MAPT/tau in these droplets induces MAPT/tau fibrillation, without LLPS of MAPT/tau. A direct interaction between RNF216 and LC3 results in sequestration of the MAPT/tau cargo within autophagosomes for autophagic degradation [208].

Huntingtin exon 1 (HTT_ex1_), associated with Huntingtin disease, is another aggregation prone protein that undergoes LLPS. Phase separation is independent of electrostatic interactions and is driven by polyQ and proline-rich regions. Lipid droplets of HTT_ex1_ convert over time into solid-like assemblies [209]. PolyQ repeats are recognized by the SQSTM1 receptor and cleared by autophagy, but only in the liquid phase separated form; fibrillar repeats are not recognized and degraded [210]. Thus, biocondensophagy is essential in preventing polyQ toxicity.

Other proteins that are associated with neurodegenerative diseases and undergo LLPS are SNCA/α-synuclein, FUS RNA binding protein (FUS), and TAR DNA-binding protein (TARDBP/TDP-43) [211,212,213,214]. For SNCA, a recent study reported that LLPS of SNCA is not a driver of its aggregation. Instead, ubiquilin 2 (UBQLN2) undergoes LLPS and catalyzes SNCA fibrillation by incorporating it into its droplets. Solidification of the UBQLN2 droplets promotes SNCA aggregation [215], a process similar to that of RNF216 and MAPT/tau. Although SNCA is targeted for removal by autophagy, this protein has a capacity to disrupt the autophagy pathway at several stages [216]. The FUS protein elicits its degradation response depending on its aggregation state. Mature acidic lysosomes accumulate near solid-like aggregates but not liquid FUS condensates. Disruption of the liquid condensates results in FUS aggregation and recruitment of functional lysosomes [217]. Whether or not liquid droplets of TARDBP are the cargo for autophagy is not completely understood. Research studies showed that chaperone-mediated autophagy can degrade TARDBP but the pathway itself is negatively affected by the TARDBP aggregates [218].

The IDR-containing P-GranuLe abnormality 1 (PGL-1) and PGL-3 and intrinsically disordered proteins such as Maternal Effect Germ-cell defective 3 (MEG-3) and ATP-dependent RNA helicase Lethal And Feminizing 1 (LAF-1) from *C. elegans* phase separate into liquid droplets. LLPS of these polypeptides is an RNA-induced process regulated by phosphorylation and by the mRNA-binding protein MEX-5 that controls accessibility of RNA [219,220,221,222]. Selective biocondensophagy of P droplets, utilizing SEPA-1 as a receptor, regulates their concentration during embryonic development of *C. elegans* [223]. A study focused on PGL granules showed that SEPA-1 promotes LLPS of PGL-1 and PGL-3, whereas the Ectopic P Granules protein 2 (EPG-2) has a capacity to convert them into a gel-like state. Analogously to polyQ, only PGL liquid droplets, not PGL gel-like granules, are the cargo for biocondensophagy [224].

## 6. Conclusions

The intersection of autophagy with IDPs and LLPS has fundamentally rewritten our understanding of cellular clearance. It is now known that autophagy initiation, cargo selection, and degradation are all directed by transient, phase-separated biomolecular condensates. However, studying how IDPs and LLPS interact with autophagy presents major experimental and conceptual bottlenecks. A major challenge in the field is developing accurate metrics to definitively prove that a specific protein forms a true liquid-like droplet within a living cell, rather than being an artificial aggregate caused by overexpression. IDPs and proteins with IDRs make up a vast portion of the human proteome. The inherent structural flexibility and functional adaptability of these entities impede targeted interventions via conventional rigid-body therapeutics. Pathological conditions and dysregulation can trigger the transition of functional liquid-like condensates into toxic, solid gel aggregates, as observed in neurodegeneration. The precise biological triggers initiating this irreversible phase transition, alongside the inability of cells to clear these rigid aggregates, remain largely undefined. To bypass these limitations, research and therapeutic developments are rapidly evolving. Rather than focusing on specific misfolded proteins, novel treatments aim to alter the physicochemical properties of the cellular interior to prevent or reverse aberrant LLPS. There is a growing reliance within the field on high-resolution tools, such as nuclear magnetic resonance, single-molecule fluorescence methods, and modern molecular dynamics simulations, to decipher the relationship between distinct amino acid sequences, multivalency, and phase separation. Researchers are unlocking “undruggable” pathological targets by designing custom molecules via generative AI that are capable of binding specifically to highly flexible IDPs. Current research is pivoting toward “intrinsic” receptors, i.e., scaffold or hub proteins within a macromolecular complex that both drive condensate formation via their IDRs and act as docking stations to selectively recruit other components for downstream processing or degradation. Ongoing studies are revealing how LLPS regulates not only standard autophagy but also the targeted sorting of cellular cargo, and how defects in these pathways contribute to pathological conditions, such as proteinopathies.

Although LLPS is a relatively recently disclosed phenomenon, our understanding of its importance for cellular function is rapidly expanding. The process of autophagy, which heavily relies on IDRs in its protein machinery, is essential for maintaining the homeostasis of functional cells. In this review, we show that LLPS is an indispensable mechanism in autophagy and involves both the core protein machinery as well as the cargo destined for degradation. Membranes from the ER, Atg9-containing vesicles, the vacuole/lysosome, endosomes, and phagophores are the sites where membrane-less separation of proteins occurs in order to engulf the cargo either directly by the membrane of the degradative organelle or through a double-membrane intermediate, the autophagosome. With a relatively small volume of research, we embarked on an intriguing journey of discoveries of how liquid protein droplets mediate cytosolic clearance or are cleared by autophagy.

## Figures and Tables

**Table 1:** Some disorder-based characteristics of the core human autophagy-related proteins.

Gene Name	Protein Name	UniProt ID	PPIDR ^a^	ADS ^b^	p_LLPS_^c^	Average p_LDDT_^d^	Protein Length
*ATG14*	autophagy related 14	Q6ZNE5	61.18	0.5940	0.8733	73.50	492
*RB1CC1*	RB1 inducible coiled-coil 1	Q8TDY2	60.23	0.5922	0.7297	72.50	1594
*ULK1*	unc-51 like autophagy activating kinase 1	O75385	59.24	0.6254	0.9977	59.41	1050
*ULK2*	unc-51 like autophagy activating kinase 2	Q8IYT8	58.59	0.5840	0.9976	58.94	1036
*BECN1*	beclin 1	Q14457	58.00	0.5509	0.5300	76.56	450
*ATG2A*	autophagy related 2	Q2TAZ0	53.10	0.5129	0.9740	67.38	1938
*ATG16L1*	autophagy related 16 like 1	Q676U5	51.07	0.4930	0.4387	83.88	607
*ATG12*	autophagy related 12	O94817	49.29	0.5289	0.6456	78.88	140
*ATG9B*	autophagy related 9B	Q674R7	48.05	0.5079	0.7625	67.94	924
*GABARAPL1*	GABA type A receptor associated protein like 1	Q9H0R8	47.01	0.4843	**0.1843 ^e^**	95.00	117
*ATG13*	autophagy related 13	O75143	44.10	0.4809	0.8633	63.84	517
*GABARAP*	GABA type A receptor-associated protein	O95166	41.88	0.4589	**0.0959**	94.94	117
*MAP1LC3B*	microtubule associated protein 1 light chain 3 beta	Q9GZQ8	40.80	0.4434	**0.1002**	91.44	125
*ATG2B*	autophagy related 2B	Q96BY7	40.66	0.4567	0.9763	65.19	2078
*ATG4D*	autophagy related 4D cysteine peptidase	Q86TL0	40.30	0.4594	0.6230	77.38	474
*ATG9A*	autophagy related 9A	Q7Z3C6	37.78	0.3904	0.4241	73.69	839
*MAP1LC3A*	microtubule associated protein 1 light chain 3 alpha	Q9H492	34.71	0.4510	0.1019	91.31	121
*MAP1LC3C*	microtubule associated protein 1 light chain 3 gamma	Q9BXW4	34.69	0.4527	0.1405	79.88	147
*ATG3*	autophagy related 3	Q9NT62	34.39	0.4174	0.2995	73.38	314
*ATG4C*	autophagy related 4C cysteine peptidase	Q96DT6	33.62	0.3751	**0.2515**	79.75	458
*PIK3C3*	phosphatidylinositol-3-kinase catalytic subunit type 3	Q8NEB9	29.31	0.3914	0.3772	83.44	887
*PIK3R4*	phosphoinositide-3-kinase regulatory subunit 4	Q99570	29.31	0.3765	0.4510	78.06	1358
*ATG4A*	autophagy related 4A cysteine peptidase	Q8WYN0	27.14	0.3320	0.3106	84.88	398
*WIPI1*	WD repeat, domain phosphoinositide interacting 1	Q5MNZ9	22.65	0.3870	0.3271	77.88	446
*ATG101*	autophagy related 101	Q9BSB4	19.72	0.3432	**0.1673**	90.12	218
*WIPI2*	WD repeat domain, phosphoinositide interacting 2	Q9Y4P8	18.06	0.3328	0.1129	76.00	454
*GABARAPL2*	GABA type A receptor associated protein like 2	P60520	17.95	0.3489	**0.1196**	94.75	117
*ATG5*	autophagy related 5	Q9H1Y0	17.82	0.3247	**0.1362**	93.25	275
*WDR45B*	WD repeat domain 45B	Q5MNZ6	16.57	0.3076	**0.1369**	94.81	344
*ATG4B*	autophagy related 4B cysteine peptidase	Q9Y4P1	13.23	0.3052	0.2045	86.19	393
*WDR45*	WD repeat domain 45	Q9Y484	11.94	0.3316	0.1460	90.50	360
*ATG10*	autophagy related 10	Q9H0Y0	11.82	0.3025	0.1266	80.19	220
*ATG7*	autophagy related 7	O95352	11.81	0.2978	0.2325	87.62	703

^a^ PPIDR values generated by PONDR^®^ VSL2. ^b^ ADS values generated by PONDR^®^ VSL2. ^c^ p_LLPS_ is the protein’s probability to undergo spontaneous LLPS evaluated by FuzDrop [156]. Proteins with a p_LLPS_ value of ≥0.60 are classified as droplet drivers capable of autonomous phase separation. At the same time, those with p_LLPS_ < 0.60 but containing droplet-promoting regions (DPRs defined by consecutive residues with residue-level droplet-promoting probabilities (p_DP_) of 0.60 or higher) are considered droplet clients that may require partner interactions to participate in condensates. ^d^ p_LDDT_ are the AlphaFold-generated per-residue confidence scores that range between 0 and 100 and that serve as a measure to evaluate the global quality of protein structural models as very high (p_LDDT_ > 90), high (90 > p_LDDT_ > 70), low (70 > p_LDDT_ > 50), and very low (p_LDDT_ < 50) [157,158]. ^e^ Bold font shows p_LLPS_ for proteins which are not related to LLPS (they have low p_LLPS_ values and do not contain IDPRs).

**Table 2:** Global classification of intrinsic disorder status in the analyzed protein sets.

Protein Set	Content of Areas in the ADS vs. PPIDR Plot (%)					Content of Quadrants of the CH-CDF Plot (%)			
	Blue	Cyan	Pink	Light Pink	Red	Q1	Q2	Q3	Q4
Autophagyome	0.00	0.00	39.39	36.36	24.24	66.67	33.33	0.00	0.00
Interactome of autophagyome	0.00	0.95	28.84	32.45	35.86	61.86	26.56	9.68	1.90
Microautophagyome	0.00	0.00	11.76	15.69	72.55	25.49	50.98	21.57	1.96
Interactome of microautophagyome	0.00	1.28	28.94	27.66	42.12	52.75	33.52	11.90	1.83
Proteome	0.41	5.07	33.67	21.01	39.84	59.13	25.48	12.31	3.08

**Table 3:** Disorder-based characteristics of the human microautophagy-related proteins.

Gene Name	Protein Name	UniProt ID	PPIDR ^a^	ADS ^b^	p_LLPS_^c^	Average p_LDDT_^d^	Protein Length
**ESCRT-0**							
*HGS*	hepatocyte growth factor-regulated tyrosine kinase substrate	O14964	71.17%	0.7368	0.9987	66.12	777
*STAM*	signal transducing adapter molecule	Q92783	63.89%	0.6263	0.8919	67.81	540
*STAM2*	signal transducing adapter molecule 2	O75886	60.76%	0.5737	0.9576	68.62	525
**ESCRT-I**							
*TSG101*	tumor susceptibility 101	Q99816	53.08%	0.5357	0.5102	82.94	390
*VPS28*	VPS28 subunit of ESCRT-I	Q9UK41	37.56%	0.4257	0.4558	91.62	221
*VPS37A*	VPS37Asubunit of ESCRT-I	Q8NEZ2	75.06%	0.6008	0.6395	76.12	397
*VPS37B*	VPS37B subunit of ESCRT-I	Q9H9H4	77.89%	0.7341	0.9585	75.31	285
*VPS37C*	VPS37C subunit of ESCRT-I	A5D8V6	100.00%	0.8876	0.9952	67.62	355
*VPS37D*	VPS37D subunit of ESCRT-I	Q86XT2	77.69%	0.7644	0.7213	79.94	251
*MVB12A*	multivesicular body subunit 12A	Q96EY5	50.92%	0.5334	0.7247	77.25	273
*MVB12B*	multivesicular body subunit 12B	Q9H7P6	44.20%	0.5066	0.6717	76.31	319
*UBAP1*	ubiquitin associated protein 1	Q9NZ09	68.33%	0.6339	0.9756	62.50	502
*UBA6*	ubiquitin like modifier activating enzyme 6	A0AVT1	18.35%	0.3274	0.2961	91.44	1052
*UMAD1*	UBAP1-MVB12-associated (UMA)-domain containing 1	C9J7I0	67.15%	0.6248	0.4202	61.44	137
**ESCRT-III**							
*CHMP1A*	charged multivesicular body protein 1A	Q9HD42	83.67%	0.7311	0.3979	77.94	196
*CHMP1B*	charged multivesicular body protein 1B	Q7LBR1	87.44%	0.7560	0.6464	80.81	199
*CHMP2A*	charged multivesicular body protein 2A	O43633	84.68%	0.7809	0.5324	75.19	222
*CHMP2B*	charged multivesicular body protein 2B	Q9UQN3	100.00%	0.8144	0.4588	79.50	213
*CHMP3*	charged multivesicular body protein 3	Q9Y3E7	92.34%	0.7718	0.6920	81.38	222
*CHMP4A*	charged multivesicular body protein 4A	Q9BY43	100.00%	0.8373	0.5161	79.12	222
*CHMP4B*	charged multivesicular body protein 4B	Q9H444	92.41%	0.7812	0.5680	78.88	224
*CHMP4C*	charged multivesicular body protein 4C	Q96CF2	96.14%	0.7816	0.7075	76.44	233
*CHMP5*	charged multivesicular body protein 5	Q9NZZ3	96.80%	0.7460	0.7315	79.38	219
*CHMP6*	charged multivesicular body protein 6	Q96FZ7	79.60%	0.7439	0.2131	81.94	201
*CHMP7*	charged multivesicular body protein 7	Q8WUX9	49.45%	0.5504	0.2843	76.00	453
*IST1/CHMP8*	IST1 factor associated with ESCRT-III	P53990	54.67%	0.5668	0.9679	72.25	364
**ESCRT-associated**							
*VPS4A*	vacuolar protein sorting 4 homolog A	Q9UN37	43.02%	0.4838	0.7784	85.69	437
*VPS4B*	vacuolar protein sorting 4 homolog B	O75351	43.69%	0.4910	0.4998	85.94	444
*VTA1*	vesicle trafficking 1	Q9NP79	39.74%	0.4635	0.6706	78.25	307
*PDCD6IP/ALIX*	programmed cell death 6 interacting protein	Q8WUM4	40.55%	0.4978	0.2523	83.62	868
*PTPN23*	protein tyrosine phosphatase non-receptor type 23	Q9H3S7	57.70%	0.6027	0.7223	69.56	1636
*USP8/UBPY*	ubiquitin specific peptidase 8	P40818	56.25%	0.5976	0.7837	71.25	1118
*STAMBP*	STAM binding protein	O95630	53.77%	0.5218	0.4213	84.00	424
**SNARE proteins**							
*SNAP29*	synaptosome associated protein 29	O95721	87.60%	0.7282	0.7691	75.31	258
*STX7*	syntaxin 7	O15400	77.01%	0.6543	0.2035	78.56	261
*STX17*	syntaxin 17	P56962	41.39%	0.4552	0.2000	69.06	302
*VAMP7*	vesicle associated membrane protein 7	P51809	13.18%	0.2900	**0.1034 ^e^**	84.31	220
*VAMP8*	vesicle associated membrane protein 8	Q9BV40	65.00%	0.4805	0.2765	89.88	100
*VTI1B*	vesicle transport through interaction with t-SNAREs 1B	Q9UEU0	81.03%	0.6453	0.4953	83.19	232
*YKT6*	YKT6 vesicular SNARE protein	O15498	27.78%	0.3855	**0.1191**	90.88	198
**Mechanistic target of rapamycin kinase complex 1 (MTORC1)**							
*MTOR*	mechanistic target of rapamycin kinase	P42345	19.62%	0.3296	0.5252	78.00	2549
*RPTOR*	regulatory associated protein of MTOR complex 1	Q8N122	23.45%	0.3524	0.6124	79.75	1335
*MLST8*	MTOR associated protein LST8	Q9BVC4	13.50%	0.3095	0.1257	91.62	326
*AKT1S1/PRAS40*	AKT1 substrate 1	Q96B36	100.00%	0.8506	0.9906	66.94	256
*DEPTOR*	DEP domain containing MTOR interacting protein	Q8TB45	47.19%	0.5245	0.7526	79.75	409
**Other proteins**							
*HSPA8/HSC70*	heat shock protein family A (Hsp70) member 8	P11142	38.70%	0.4531	0.3211	88.31	646
*NBR1*	NBR1 autophagy cargo receptor	Q14596	62.11%	0.5697	0.8908	58.59	966
*TAX1BP1*	Tax1 binding protein 1	Q86VP1	78.96%	0.6559	0.7498	75.94	789
*NCOA4/ARA70*	nuclear receptor coactivator 4	Q13772	56.19%	0.5290	0.7727	55.97	614
*SEC62*	SEC62 preprotein translocation factor	Q99442	56.89%	0.5857	0.9856	65.19	399
*CALM1*	calmodulin 1	P0DP23	69.13%	0.5353	**0.1763**	85.25	149

^a^ PPIDR values generated by PONDR^®^ VSL2. ^b^ ADS values generated by PONDR^®^ VSL2. ^c^ p_LLPS_ is the protein’s probability to undergo spontaneous LLPS evaluated by FuzDrop [156]. Proteins with a p_LLPS_ value of ≥0.60 are classified as droplet drivers capable of autonomous phase separation. At the same time, those with p_LLPS_ < 0.60 but containing droplet-promoting regions (DPRs defined by consecutive residues with residue-level droplet-promoting probabilities (p_DP_) of 0.60 or higher) are considered droplet clients that may require partner interactions to participate in condensates. ^d^ p_LDDT_ are the AlphaFold-generated per-residue confidence scores that range between 0 and 100 and that serve as a measure to evaluate the global quality of protein structural models as very high (p_LDDT_ > 90), high (90 > p_LDDT_ > 70), low (70 > p_LDDT_ > 50), and very low (p_LDDT_ < 50). ^e^ Bold font shows p_LLPS_ for proteins that are not related to LLPS (they have low p_LLPS_ values and do not contain IDPRs).

## Data Availability

No new data were created or analyzed in this study. Data sharing is not applicable to this article.
